# Experiences of Working with the Tobacco Issue in the Context of Health Promoting Hospitals and Health Services: A Qualitative Study

**DOI:** 10.3390/ijerph8020498

**Published:** 2011-02-15

**Authors:** Siw Carlfjord, Margareta Kristenson, Malou Lindberg

**Affiliations:** 1 Division of Community Medicine, Department of Medical and Health Sciences, Linköping University, SE-581 83 Linköping, Sweden; E-Mails: siw.carlfjord@liu.se (S.C.); margareta.kristenson@liu.se (M.K.); 2 Local Health Care Research and Development Unit, County Council in Östergötland, S:t Larsgatan 9 D, SE-582 24 Linköping, Sweden

**Keywords:** health-promoting hospitals, setting-based health promotion, smoking cessation, tobacco, public health, anti-tobacco policy making

## Abstract

The worldwide Health Promoting Hospital and Health Services (HPH) network was initiated by the World Health Organizations in the late 1980s. The goal of the network is to change the focus of health services from curing patients to also embrace disease prevention and health promotion. In Sweden the network started in 1996, and involves mainly hospitals and primary care. The network members collaborate in task forces, one of which is working on the tobacco issue. There is limited evidence on the value of working within an HPH organization. The aim of this study was to investigate the experiences of members of the Swedish HPH network tobacco task force. Focus group interviews with task force members were analyzed using implementation theory. Three themes, overall experiences of working with tobacco issues, experiences of working with “free from tobacco in connection with surgery”, and experiences of work in the HPH tobacco task force, emerged from the interviews. The results show that working with the tobacco issue in the context of health-promoting hospitals and health services met with difficulties involving the following important factors: evidence, context, facilitation and adopter characteristics. Leadership, one contextual factor, at national and local level, seems to be crucial if the work is going to succeed. The tobacco task force of the HPH network is an important facilitator supporting the task.

## 1. Introduction

The international Health Promoting Hospital and Health Services (HPH) network was initiated by the World Health Organizations (WHO) in the late 1980s. The aim of this visionary network is to change the focus of health services from curing patients to also embrace disease prevention and health promotion [1,2]. Today the organization includes 30 countries, mostly in Europe, but also Australia, Canada, South Africa, Taiwan, and the United States.

The Swedish HPH network started in 1996, and today it includes about 75% of hospitals and primary care centers in Sweden. It is a non-profit organization, mainly financed by membership fees. In 2009–2010, the organization was also supported by government grants. The main criterion for membership is a desire to develop a more health-promoting health service within the organization. Membership therefore implies a documented management decision that the hospital/health care organization will act in this direction [3]. One opportunity for the members to work in this direction is to collaborate in the various task forces on specific topics. Today (November 2010) there are 10 different task forces covering different areas such as alcohol, food and tobacco issues. These task forces are one of the central means for the HPH network to implement the vision. They operate according to work plans developed by members of the taskforce but which are also decided on by the general assembly of the network, led by a chair. The tobacco task force started in 2005 and was one of the first task forces in the Swedish network. The goal of this task force is that the member organizations are united in becoming tobacco free, *i.e.*, work together towards a tobacco-free health care, and, in cooperation with public institutions, voluntary organizations and other community partners, work for a tobacco-free environment and society. Since 2010 Sweden is also a member of the European Network of Smoke-free Hospitals (ENSH).

One specific issue in this area is smoking cessation in connection with surgery. The evidence on reduced complications and enhanced recovery after surgery among those who ceased smoking is increasing [4–6]. In an RCT from 2002, Møller *et al.* [4] found that patients in for hip or knee replacement surgery who were provided with smoking intervention had less postoperative complications and shorter lengths of stay than the control group. In another study, Lindstrom *et al.* [5] found a relative risk reduction of postoperative complications of 49% among patients who had quit smoking in connection with surgery. Nasell *et al.* [6] showed that among smokers, the odds of having a complication were 2.51 times higher than among those who had quit smoking before surgery. Therefore, in recent years, the tobacco task force has focused on this issue. This work has included the development of a patient information leaflet advocating the benefits of being tobacco free in connection with surgery in local health care settings. In 2009, the project “Free from Tobacco in Connection with Surgery” began. The first part of the project involved a survey of common practice in all hospitals in Sweden, showing that a great number of the participating hospitals had no or very limited experience of working with the issue [7]. The second part of this project is the present qualitative study.

It is well known that the transmission of evidence-based medicine is a slow and unpredictable process and there is also a lack of knowledge about how to put health-promoting concepts into practice in health care settings [8–10]. The HPH network could be an important arena for the development of a knowledge base in the health promotion field for experts and scientists, and could facilitate implementation of evidence-based medicine in practice [11]. Implementation science is an expanding field and a number of models and frameworks have been presented. One model often used to evaluate implementation is the Promoting Action on Research Implementation in Health Services (PARIHS) framework [12,13]. The framework suggests that implementation success is a function of the nature and type of evidence, the qualities of the context, and the way the process is facilitated [13]. Another important factor, not mentioned in the PARISH framework but stressed by other authors in the field of implementation theory, is the adopter characteristics [14].

Researchers have been discussing the nature and progress of the European HPH movement, and conclude that more evaluation is needed to measure its impact [15,16]. In a recently published literature review, the authors found limited evidence for the value of working under an HPH organization. The main reason was that few studies have been performed, and the authors concluded that more rigorous research on HPH and dissemination of results is needed [17]. The aim of the present study was to investigate the experiences of members of the Swedish HPH network tobacco task on work with tobacco issues, in the light of implementation theory.

## 2. Method

### 2.1. Study Design

The study was conducted using a qualitative method with focus group interviews. All the interviews were performed by the same moderator (KB) and an assistant (ML). The role of the assistant was to make notes, and if necessary, ask complementary questions. After each interview, the moderator and the assistant discussed what emerged during the interview. These discussions were taken into account in the analysis of the interviews.

### 2.2. Participants and Data Collection

An invitation to participate in interviews was sent to the 27 registered members of the HPH tobacco task force in May 2009. Fifteen members representing county councils, hospitals and primary care facilities from the north to the south of Sweden agreed to participate. Three interviews took place with 2, 5, and 8 individuals participating. The interviews, which lasted between 1.28 and 1.41 hours, were recorded on tape and transcribed verbatim, including notations of non-verbal expressions such as silence and laughter. Two of the interviews took place in Stockholm (central Sweden) and one in Helsingborg (southern Sweden), in June and July 2009. Among the participants were three physicians (two in pulmonology and one in orthopedics), eight nurses (two process managers, one manager at an orthopedic clinic, one manager at a pulmonary clinic), one statistician working as a process manager and three public health coordinators. Eleven participants were women and four were men. The time they had been members of the tobacco task force group varied from several years to less than 1 year.

The interviews started with the moderator asking some background questions, and then the moderator asked the respondents to recount and reflect on their work with the tobacco issue, in particular the issue of promoting smoking cessation in connection with surgery. During the interviews the respondents were encouraged to speak freely about the topic and the interviewer asked follow-up questions such as “Can you tell me more about that?” *etc.*

### 2.3. Analysis

A manifest qualitative content analysis as described by Graneheim and Lundman [18] was used. The narrative data were handled in a systematic way with the goal of extracting experiences and reflections from individuals as well as from the whole group. The interviews were read and re-read several times and meaning units were identified by one of the authors (SC). Early in the analysis three overall themes emerged and meaning units were sorted into the different themes. The condensed meaning units were coded and sorted into categories by two of the authors (SC, ML). Codes, categories and subcategories were discussed by the authors until consensus was reached. Back and forth movement between the whole and parts of the text was an ongoing process in the analysis.

### 2.4. Ethical Issues

The study involved only staff members in health service; no patients were involved. The data collected were handled confidentially so that no individuals can be identified in the results. According to Swedish law, the act concerning the Ethical Review of Research Involving Humans (SFS 2003:460) from the Ministry of Education and Cultural Affairs, the present study does not require ethical approval.

## 3. Findings

The participants interacted in a positive way at the interviews and helped each other to relate to the issues raised. There was no disagreement among informants within groups, but rather a high degree of consensus. Neither were there any discrepancies in opinion between the three groups, even though different issues dominated the discussion in the different interviews. Despite differences in group size, all groups acted in a dynamic way. Local examples of tobacco prevention in general and with regard to stopping tobacco use among patients in connection with surgery were described, illustrating that some hospitals and county councils have made some progress in this area; work has not advanced so far in other settings. Three themes, overall experiences of working with tobacco issues, experiences of working with “free from tobacco in connection with surgery”, and experiences of work in the HPH tobacco task force, emerged from the interviews. The themes, categories and subcategories that were deduced from the interviews are described in Tables 1–3 together with quotations to support the findings. Quotations are selective and illustrative.

### 3.1. Overall Experiences of Working with Tobacco Issues (Table 1)

#### 3.1.1. General experiences

Looking at the development over time, the informants expressed the view that public health issues in general are discussed more often in hospital care now than they were before. In all groups, this was commented on and seen to be a result of the work on tobacco prevention in Sweden done by one influential member of the tobacco task force. Even though the tobacco issue is considered important in hospitals and in primary health care (PHC), the informants feared that health care workers now believe that the tobacco problem has decreased in society. They were concerned that there are still many young people who are smoking, and felt a responsibility to work with the tobacco issue in health care. The informants stated that it is very important that both PHC and hospitals work actively and provide help with smoking cessation. The vision is to make asking about tobacco use a routine question, and to provide support to everyone who needs help to quit smoking. Informants described a hope that tobacco be discussed more throughout society, and that research and policy will cooperate to eliminate tobacco use in the future. All groups agreed that there is enough evidence to support working against smoking tobacco but that there is a lack of evidence about the dangers associated with using snuff. The informants stressed the value of research and evidence to convince different staff categories about the importance of the tobacco issue. It was questioned why physicians, requesting evidence in all situations, do not act according to the evidence available.

#### 3.1.2. Facilitators

A number of important facilitators were mentioned by the informants. Most important seemed to be that managers, politicians and policy makers support preventive work made by staff in clinical practice. Policy documents, in terms of guidelines and implementation plans, were also mentioned as crucial for success. Regarding incentives, some advocated legislation and punishments; others thought that following of guidelines or a policy must be done on a voluntary basis. Some keywords were proposed for marketing the tobacco issue; e.g., involvement, patient security, self-care and environment. A system with indicators that would help to follow up the tobacco issue and evaluate the results of interventions was requested.

#### 3.1.3. Barriers

The informants described several barriers to successful work against tobacco use in health services. Leadership, e.g., politicians or managers who are not interested in or supporting the issue were seen as an important obstacle, as well as politicians or managers who are themselves tobacco users. Ignorance due to lack of knowledge among clinicians was mentioned, as well as uncertainties about how to tackle the problem. Other barriers are the lack of structure at local level and that the digital medical record system is not adapted for documentation or follow-up of tobacco prevention. This latter problem seemed to be a major concern, and the participants anticipated a common digital system to be used on a national level. A high level of inertia at the local level was also seen as a barrier.

### 3.2. Experiences of Working with “Free from Tobacco in Connection with Surgery” (Table 2)

#### 3.2.1. General experiences

Concerning development over time, the informants recalled how the issue of smoking cessation before surgery was raised some years ago when a Danish study on the evidence for its effects on postoperative complications was published [4]. Since then, this issue has been discussed in Sweden, especially in the HPH network, and, according to the informants, it is perceived today as an important task in primary care, hospital care, and among dentists; there is also great interest among microsurgeons and vascular surgeons. Regarding priority, the informants stated that this question is perceived as very important among clinicians, especially vascular surgeons, and among policy makers. One reason for this is the evidence of reduced number of complications, and thereby immediate economic gains associated with patients being free from tobacco in connection with surgery. Informants suggested that this could influence implementation and they also suggested that there is now enough evidence supporting the task. Some informants mentioned positive experiences from their own clinical practice. A vision was described that all patients would always be free from tobacco in connection with surgery; one informant compared this vision with the fact that smoking is no longer allowed on board an aircraft; this was not expected some years ago, but now it is widely accepted.

#### 3.2.2. Facilitators

One important factor that was perceived as facilitating the work on being free from tobacco in connection with surgery was leadership, in terms of decisions made at a high management level. It is not enough that clinicians are aware of the benefits; policy makers and managers also have a crucial role. Guidelines on a national level were also mentioned as an important factor. The informants thought that a prerequisite for a hospital that wants to work with the tobacco issue in connection with surgery is that there is a non-smoking policy within the hospital—a matter of credibility. According to the informants, knowledge about the issue has to be spread among clinicians, and it is also very important to repeatedly inform patients. Information material, specifically the patient leaflet produced earlier by the HPH network, was seen as a valuable tool. The most important issue that evolved in the discussions was a matter of process, to have a continuous chain of care in which general practitioners in PHC inform the patient and hand over the leaflet, surgeons at the hospital give the same information and nurses or other staff members who see the patient when they leave the hospital follow up and support the decision to quit tobacco use. If there is another follow-up visit in PHC, the issue should be raised once again.

#### 3.2.3. Barriers

Important barriers mentioned were opinions among staff in the local setting, especially reluctance because many staff members still use snuff or still smoke. The ongoing discussion about whether only tobacco smoking should be addressed, or if cessation of snuff use is also important, was seen as a barrier to success. Lack of knowledge among staff members was mentioned by the informants as a barrier, and more education about the issue was suggested. Another perceived obstacle mentioned in the interviews was information difficulties, such as observations that patients do not read the information provided. The lack of a digital medical record system adapted for the work and the lack of the possibility of combining different databases in order to identify associations between tobacco use and complications after surgery were also seen as obstacles. The medical record system was also mentioned with regard to the possibilities for follow-up to evaluate results at the individual and group levels. Follow-up was an issue that was mentioned as being very important for the task, but something that is still not working appropriately. Another barrier mentioned was inertia; the fact that it takes time to implement change in health care settings seems to produce a certain level of frustration among the informants.

### 3.3. Experiences of Work in the HPH Tobacco Task Force (Table 3)

#### 3.3.1. General experiences

Informants claimed that, with regard to development over time, the tobacco issue is seen as a cornerstone in the HPH network and that they always thought that tobacco was a symbolic issue. The informants described their personal feelings about the importance of working against tobacco use at the local hospital and being a part of the tobacco task force. The structure of, and the economic conditions for, the work of the task force have developed over time, which is regarded as positive by the informants. With regard to goals and visions, the informants expressed a wish for more cooperation with other task forces within the HPH network. They also report the hope of being able to influence medical education regarding the tobacco issue. With regard to recruitment to the task force, which is based on personal interest in the issue, the informants called for a mix of experts, administrative staff, and people representing different areas of the health services.

#### 3.3.2. Possibilities

According to the informants, the HPH network and the tobacco task force are considered very important on a practical level, as a forum for knowledge and experience exchange, a place where to discuss policy and guidelines and with the ability to produce good information material. At the emotional level the group is perceived by informants as very supporting, and attending the meetings is seen as a source of strength and energy. Informants expressed that knowing that there is a network supporting them gives them strength to keep up the work at the local level, and makes it easier to argue about the importance of the issue. When an organization becomes a member of the HPH network, this gives legitimacy to the work and support to their representatives in the task force. This support seems to be important for the informants, who also perceive that being a task force member gives a certain status, not least in the local setting.

#### 3.3.3. Challenges

One problem mentioned by the informants was the high number of passive members, *i.e.*, those who put their names on the list but never attend the meetings. A higher level of activity would open up new perspectives. There was a discussion about a certain level of participation required to remain a member. Discrepancy in experiences from the different hospitals/PHCs was also seen as a problem. Those who have been working with the issue for some time feel they cannot make any advances, because new members tend to discuss how to start, and the work cannot move forward. Some informants still feel that the structure has to be better, even though it has developed. Resources were discussed as a challenge. More time for work in the group would make it more effective; today it is very hard to achieve the goals that have been formulated for the group. Better economic resources have provided opportunities to develop the work.

## 4. Discussion

### 4.1. Methodological Considerations

This study was performed as a qualitative study in order to investigate how members of the HPH tobacco task force have experienced working with tobacco issues, facilitators and barriers, *etc.*, and present the results in the light of implementation theory. Using a qualitative method has the potential to add information that would not be achieved in a survey-based study, and we believe this expands knowledge on the subject. We chose a purposive sample, with the goal of delving deeper into topics and perspectives important to this specific issue. Thus, finding the most experienced informants was a prerequisite for the study. We are confident that we met members with a wide range of experience in this field and that this led to emergence of a broad perspective. The fact that one of two authors who analyzed the text did not participate in the interviews could be seen as a weakness, but could also be considered a strength. However, the interviews were recorded on tape and transcribed verbatim, and the analyses performed were manifest; it is our opinion that this weakness was controlled for, and that the study results are trustworthy.

### 4.2. Findings

Three themes arose from the interviews: working with tobacco issues in general, working with the concept “free from tobacco in connection with surgery”, and being a member of the tobacco task force. All three themes could be seen as different aspects of the experience of working with the tobacco issue in the context of HPH. They can also be seen as three aspects of implementing tobacco prevention in ordinary clinical work. Therefore, the results are discussed in the light of findings from implementation research. We have chosen the PARIHS framework, a tool developed for evaluation of implementation of evidence into practice, which suggests that implementation success is a function of evidence, context and facilitation [12,13], but also added adopters [14,19], which are also of great importance in an implementation process. Our findings are discussed on the basis of these four factors. The different factors are treated separately, but undoubtedly there are important links between them, as also stressed in the PARIHS framework [12].

#### 4.2.1. Evidence

Our informants stated that there is a strong evidence base for working with tobacco issues, not only in general but also specifically in the context of a patient presenting for surgery. Informants were concerned about lack of evidence of the dangers of using snuff, which, to them, seems to hinder the broad implementation of working against tobacco in all forms. However, it is known from implementation studies that a strong theoretical evidence base is an important, but not sufficient prerequisite for a successful implementation [12].

If follow-up, for example regarding complications after surgery, could be performed in an easy way, the evidence base for efficacy would be stronger. However, the lack of structure in digital systems, mentioned by the informants, leads to difficulties in documenting. Evidence of economic gains is also important to convince policy makers about the urgency of the issue. A strong evidence base converted into national guidelines would be very supportive in executing the task. The Swedish guidelines on disease prevention in health care services, recently published, do have very strong recommendations on providing advice about smoking cessation in connection with surgery [20].

#### 4.2.2. Context

Looking at context, the informants perceived that the tobacco issue is now more important in society than before. They also expressed that it is a responsibility of health services, both in hospitals and in PHC, to address the issue. Why then, is this not always done? One possibility is that the feeling of being responsible for prevention is not shared by all colleagues [21]. One core component seems to be the lack of a supportive leadership at the local level, but also at higher levels. Leaders have to support the task, and also provide facilitating structures, such as joining the HPH network. However, joining the network does not seems to be enough if leaders at all levels do not agree with the importance of the task. The importance of leadership is often mentioned in the literature regarding change management. Van de Ven states that, to establish structures and systems that facilitate innovation, the institutional leadership is crucial [22]. Highly educated and cosmopolitan hospital administrators have positively influenced the adoption of administrative innovations [23]. Leadership is also important to overcome the barriers of a medical record system that is not adapted for the task. To influence the IT companies that provide these technical solutions requires decisions at a high management level, and perhaps also at national level.

Another factor in the area of context is the perceived inertia that informants mentioned as a barrier, both in working with the tobacco issue in general, and working to stop tobacco use among patients undergoing surgery. The health care system is a huge organization, far from unitary in character, and there are many levels that have to be influenced to achieve change. Probably HPH has to invest more in lobbying, to create a context that better supports the aims of HPH and its members. Again leadership is important to support the process, but there still might be reluctance among health care professionals to change behavior. This is discussed further in Section 4.2.4 on adopter characteristics. Despite the perceived inertia, the informants in our study also expressed their vision for work with the tobacco issue. If these visions were shared by managers as well as professionals in the health services, the implementation would be much improved. Visionary people, sometimes called opinions leaders, have been found to play an important role in an implementation process, particularly in groups of highly specialized staff [24].

#### 4.2.3. Facilitation

The third issue in the PARIHS framework is the way the process is facilitated. In our study the informants themselves were supposed to be facilitators in their particular settings. However, they also mentioned other ways that could have facilitated the process, e.g., policy documents and incentives. The use of incentives and reinforcement has, on occasions, been found to be successful in improving the use of research in clinical settings, but the evidence supporting these strategies is mixed and limited [25]. Establishing a continuous chain of care was another important issue mentioned in the interviews, especially regarding tobacco cessation in connection with surgery. This chain of care requires elements of structure (staff and educational resources, information materials, *etc.*), process (cooperation between hospitals and PHC and other actors in society, e.g., politicians) and results (based on the digital medical record system), according to the quality framework provided by Donabedian in 2005 [26]. It seems that there are failures in all these elements as things stand today, but the information material that has been produced by the task force is one way of providing structure, and is also perceived as very important by the group members.

Working in the task force was perceived very positively by the informants in our study. The informants had visions of cooperating with other task forces, working with other lifestyles and, even, visions of influencing medical education in order to facilitate the implementation of a more health promotion-oriented health care system. If financial resources are provided, and if participants who are not only interested in the subject but are also influential on a management level could be recruited, the group could probably become an important facilitator for the implementation of tobacco issues in health services in the future. According to Rycroft-Malone [12] there should be appropriate facilitation of change with input from skilled external and internal facilitators. Members of the task force could act as both internal and external facilitators.

#### 4.2.4. Adopters

With regard to the adopter characteristics, one barrier to implementation perceived by the informants is a certain level of ignorance due to lack of knowledge among health care professionals. More education for staff members at the local level is suggested. Hopefully, this will also change opinions among staff who are reluctant to work with the issue, sometimes because they themselves are still smoking or using snuff. However, knowledge is not the only way to achieve behavior change among professionals. There are a number of theories from behavioral science that could be used to explain why it is so hard to implement change in clinical practice; some of these are discussed by Grol *et al.* [27]. In their book, *Improving Patient Care*, the authors provide an implementation model and include an analysis of the target group.

## 5. Conclusions

The difficulties in implementing work on tobacco issues in the context of health promoting hospitals and health services include several important factors: evidence, context, facilitation and adopter characteristics. These difficulties arise in tobacco work in general, and in advocating tobacco cessation in connection with surgery. Leadership, one contextual factor, at national and local levels, seems to be crucial if the work is going to succeed. The tobacco task force of the HPH network is an important facilitator supporting the task. Knowledge gained from this study will be taken into account in future HPH work, e.g., by producing educational material targeted to patients and staff members for use in the local setting.

## Figures and Tables

**Table 1.** Theme: overall experiences of working with tobacco issues.

**Category**	**Subcategory**	**Quotation**
General experiences	Development over time	I believe it has become more accepted that … health is also our concern …. not just curing diseases and such … (IV, group 2)
	Responsibility	… open care units work more with primary prevention than hospitals, but not everything can be left to open care units just because of that, so it’s a matter of putting it on a hospital level as well (III, group 2)
	Vision	… that our grandchildren shall be born into a tobacco-free society (IV, group 3)
	Evidence base	… it’s seldom that there is so much unequivocally clear evidence for … how good it is (VII, group 2) There’s so much evidence for working with tobacco issues, … that … no one questions it (I, group 1) It’s still remarkable that … the Swedish medical profession … that is so, that demands so much evidence, but still finds it so terribly difficult … to comply… it’s grotesque and embarrassing that it’s so difficult and sometimes one feels that this evidence, ah … the demand for it is a constant obstacle … (I, group 2)
Facilitators	Support	… if the management is with us, then it’s much easier (IV, group 3) … we now have a county council director interested in prevention … we’ve missed that for many years. Such things also make a difference (II, group 1)
	Policy documents	… and if you have a policy and a decision on how to implement this then you can, then it’s no problem for there are no obstacles … then it’s just to solve the problems (IV, group 3) And getting it, just as you say, getting it into the health care agreement. That this is something basic, that must get done! (II, group 1)
	Incentives	And if … auditors then discover that the hospital directors or hospital… here … don’t do the job … then there will be a reprimand from the auditors (I, group 2) But it feels wrong … in some way, so that it’s … it’s much easier if you can get people to work with you rather than forcing them to work with it (I, group 1) And by putting their name on the employment contract, they are also committed to follow … ah laws, regulations, policies … and … uh … ask them if they are aware of what can happen if they don’t do this [such as personnel not following the hospitals smoke-free policy]… and that, that I say that they have to see this as, as a verbal warning that they are absolutely forbidden to do this and they’d better follow this … next step is a written warning which can gradually lead to getting fired (VI, group 3)
	Marketing	It’s when you say this with headings, that yes, with the entire concept of “good care”, to use those headings and use them as well (VI, group 2)
	Follow-up	The hospital hasn’t followed up the numerical values, and it’s important to get them into … eh, the budget and the long-term plan (III, group 2) It can be written in the agreement that it is to be carried out, but if it is not followed up and evaluated, then it will not get done … what can I say, it won’t be carried out in the manner conceived (II, group 1)
Barriers	Leadership	… it is primarily a question of management, I think that keeping the personnel smoke-free depends heavily on the head of the ward … if that person smokes then it’s immediately difficult … (IV, group 2) … as a matter of principle the manager feels that these things have no place at all at the hospital but should be dealt with in primary care. So he says categorically no, despite the fact that he has an organization that works correctly and properly. That’s also the way it can be (II, group 1)
	Lack of knowledge	I don’t believe that knowledge is as widespread among … our colleagues as we believe … (VIII, group 2)
	Structure	But I feel that we still need to structure our work … especially at the hospital, I think (I, group 1)
	Medical record system	But it’s also remarkable that in our extensive medical records system […] that you cannot perform a free text search (V, group 2)
	Inertia	But it, it is sluggish … very sluggish in [name of town], it’s a big hospital … and to get them to care about this small part is … (V, group 3)

… = Hesitation,

[ ] = Authors explanation,

[…] = Some words left out.

**Table 2.** Theme: experiences of working with “free from tobacco in connection with surgery”

**Category**	**Subcategory**	**Quotation**
General experiences	Development over time	That question has been around ever since the theme group began … and was one of the first parts (I, group 1)
	Priority	On the other hand, medicine, pulmonary medicine … that entire package and surgery and … primarily vascular surgery, they say Hallelujah, that’s what I want! (II, group 1) That’s what they [managers] understand, less infections—fewer days of care, money, money … and then in connection with operations, that they understand even if they don’t understand the evidence behind it (VII, group 2)
	Evidence	It becomes more and more current the more … ah … articles that are produced, the more research that is presented, the importance of stopping in connection with an operation and not only just before it (III, group 3) The latest studies show that improvements can be seen even if they stop smoking 24 hours before [an operation] (IV, group 2)
	Vision	So, it was utopian in that you weren’t to smoke between New York and Paris … damn, that is obvious today, where then, is the utopia? (IV, group 3)
Facilitators	Leadership	Yes, but I can’t convince my colleagues to work in this way if I have no support from the management … who have decided that we are to work that way … it doesn’t work … if you don’t have it … then you can’t, no … (IV, group 3)
	Guidelines	And now come the guidelines … from The National Board of Health and Welfare […] and there comes … what we were talking about, being a part of those guidelines (I, group 2)
	Credibility	In order to be able to give them the best results[…] you need … credibility … as personnel, you have to be a good example, and to do this you have to be smoke-free at the hospital […] and have a tobacco-free policy (IV, group 3)
	Knowledge	That healing improves, and that maybe people are aware of this, but what percentage, and what does it imply in hospital days and what are the implications for costs and suffering …? (IV, group 2)
	Information material	We have together produced … material ,… that leaflet looks more or less identical […] but it has been helpful, I think, for a line of argument … that we have discussed … what information is to be in it so that everyone can understand it (IV, group 2) … some type of short program for doctors to show in a PowerPoint presentation or on the computer or something, just concerning a smoking stop before surgery, the importance of it (I, group 1)
	Process	Very important that when they come home, they should be automatically contacted [by PHC], so they can get this information there first, then further information at the hospital, when you go home so you get someone [from primary health care] to be in contact with afterwards (I, group 1)
Barriers	Opinions	But from what I’ve seen there, if you have a smoking nurse or nurse’s aid having this conversation, they skip this question. They don’t mention it (II, group 2) And it’s a question of credibility. If I’m going to be operated on and the person opposite me is telling me to quit smoking while I can smell the smell of old cigarettes on them … I’m not going to accept their message (II, group 1) An orthopedic surgeon lectured and pointed out time and time again it was a question of cigarettes … cigarettes and not snuff (III, group 3)
	Lack of knowledge	Because we need to raise the level of knowledge so that everyone really knows this properly (I, group 1)
	Information	You look, and see you have a date for surgery, and that date, that is what is interesting. If it’s accompanied by something more it’s easy to overlook it, some read, some read everything they’re sent, but some don’t read at all (II, group 2)
	Medical record system	… and it’s a very slow and old fashioned system to get in follow-ups and parameters that weren’t there before (II, group 2)
	Follow-up	Yes it’s a defect in … all the work we do, the lack of follow-up … we are … very energetic … or fairly energetic when starting something new, but the follow-up is … unfortunately neglected … if there is no way to build it in from the beginning in some way (I, group 2) Yes, when you’ve put in so much work and then there’s no one who requests it … then it’s no use (V, group 2)
	Inertia	This was some time in October 2008 [the heads of the clinic decided to test the question]… we are not to believe it will happen fast (II, group 1) Then there has been a load of discussions, but it’s like lice on a stick of tar … which is what it’s about … we shouldn’t believe it will happen quickly (II, group 1)

… = Hesitation,

[ ] = Authors explanation,

[…] = Some words left out.

**Table 3.** Theme: experiences of work in the HPH tobacco task force.

**Category**	**Subcategory**	**Quotation**
General experiences	Development over time	I believe that the tobacco task force existed before we began, so to speak, working in a more structured manner … theme groups in that way. It felt like there was a great need and there was a foundation […] in the network … for health promotion you must work with the issue of tobacco (I, group 1) The work was not so structured initially, but was more of a network, and we met, but it has changed […] has gained a structure (V, group 3)
	Goals	It is to attempt to influence medical training. To raise these questions, to lift the importance of tobacco-preventive measures (II, group 1) So we can cooperate with other theme groups in some way with this, in our job as … a tobacco theme group (IV, group 2)
	Recruitment	Some things we need to think of concerning theme groups in general […] that sometimes it is the specialists, those who are dedicated, who come to the theme groups, because those are the ones they send. But they have no proper grip of HFS work perhaps, and they have no idea of how to work within other structures […]so there is therefore a need for … a mixture and a blend (I, group 1)
Possibilities	Practical	I see this more as networking, where we meet regularly and can exchange experiences (II, group 1) It is the role of the theme group to update and remind all hospitals that now something new has come, new facts to be updated *…* (VI, group 2)
	Emotional	Yes, I believe we have some everyday knowledge of this … psychologically this is also considered … status … to be part of a network, to participate in this way of reasoning (II, group 1) It provides an unbelievable shot in the arm for work, and when you feel that, damn, this is taking so long, then suddenly, you get energy and have the strength to carry on a while longer (I, group 3)
Challenges	Participation	Because there are so many who are in the network who haven’t participated in a single meeting (II, group 1)
	Discrepancy in experience	It is a huge span for how far we have come with tobacco issues and therefore it is very difficult to, ah … get anywhere with one single question (II, group 2)
	Structure	In the short time I have participated, the schedule and subjects for discussion in the theme groups have not been determined beforehand … when you go there you don’t know the subject to be discussed […] the working method has not yet been established (II, group 1)
	Resources	Then it’s also because we have gotten more money for the network, so we have had possibilities to develop our work in another way (III, group 3) … but then you come home to your little cave and there you have … .not even had the time to do what you agreed to do for the network, there are … no margins left in the system (I, group 2)

… = Hesitation,

[ ] = Authors explanation,

[…] = Some words left out.
